# Internet-of-Things-Enabled Smart Bed Rail for Application in Hospital Beds

**DOI:** 10.3390/s22155526

**Published:** 2022-07-25

**Authors:** Solomon Ould, Matthias Guertler, Pavlos Hanna, Nick S. Bennett

**Affiliations:** 1 Centre for Advanced Manufacturing, Faculty of Engineering and Information Technology, University of Technology Sydney, Ultimo, NSW 2007, Australia; matthias.guertler@uts.edu.au (M.G.); pavlos.hanna@uts.edu.au (P.H.); 2 Radio Frequency and Communication Technologies (RFCT) Lab, Faculty of Engineering and Information Technology, University of Technology Sydney, Ultimo, NSW 2007, Australia

**Keywords:** IoT, hospital, LoRaWAN, wireless, offline LoRaWAN, local LoRaWAN, power analysis

## Abstract

This article presents an atypical offline based LoRaWAN application for use in hospital settings, where the ability to maintain network connectivity during internet connection disruption is paramount. A prototype bed rail is demonstrated, providing advanced functionality compared to traditional bed rails. The manufactured prototype provides data to a nurses station reliably and operates under battery backup. The power consumption of the system under different transmission intervals was tested, allowing appropriate battery sizing for different applications to be specified accurately. It is expected that a single LoRaWAN gateway will be able to cover bed rails across an entire modern hospital, allowing minimal infrastructure cost to implement the device or application in a rapidly deployed field hospital.

## 1. Introduction

Hospitals, rehabilitation centres, nursing and retirement homes worldwide depend on their medical staff to ensure supervision and safety of their patients. Under times of heavy workload, additional stress on medical staff can reduce the time available to focus on individual patients [1]. A specific risk exists around patients leaving their beds. Aside from potential injuries from falling to the ground, subsets of patients, e.g., dementia patients, pose additional risk, since leaving their room and wandering around the facility can result in them getting lost or walking into potentially dangerous areas [2].

Therefore, it is critical for medical staff to detect as early as possible when a patient tries to leave their bed, ideally even before they have left it. Ethical and data privacy issues limit the range of feasible solutions and exclude e.g., video monitoring. Thus, a solution is required that allows for unobtrusive and ethical monitoring of patients. Solutions also need to support flexible use of hospital beds in different areas of a facility, not require extensive and expensive hardware, not interfere with existing, often strained, infrastructure like WiFi, and allow for secure and reliable data capturing and transfer. Bed rails (sometimes called safety rails or side rails), positioned on the sides of beds in care settings, are controversial. These are specially designed rails that in the ‘up’ position provide a deliberate obstacle to anyone wishing to enter or, more likely, leave the bed. In the ‘down’ position, the rail retracts and the obstacle is removed. On the one hand, bed rails provide an effective barrier to preventive vulnerable patients falling from beds onto the adjacent floor and sustaining injuries [3]. On the other hand, a new subset of bed rail-induced injuries and deaths have occurred due to the use of bed rails [4]. Another criticism of bed rails is that they can leave those in the bed feeling trapped [5]. This is reinforced when bed rail standards require beds to have two-handed release and are deliberately difficult for those in the bed to retract. A recent solution to the use of bed rails has been the development of the floor bed, where the bed can be lowered almost entirely to floor level [6]. This prevents injuries when an occupant falls from the bed, while the beds are often equipped with floor sensors to identify when person has fallen. While this removes the need for a bed rail, it does not always alleviate the feeling of being trapped, since physical disability and old age often means a patient cannot lift themselves from the floor level. The smart bed rail proposed here is aimed at reaching a compromise between the traditional bed rail limitations (patients being trapped in the bed) and the recent alternative lowering beds (patients being trapped on the floor) by using smart technology to augment a bed rail that could be operated (up/down) more easily, i.e., potentially operated by the patient themselves. In granting this extra freedom to the patient, an IoT-enabled rail is used, which measures the position of the bed rail and communicates this wirelessly to a monitoring platform, e.g., a dashboard, to inform a nurses station that the bed rail position has changed. This is beneficial in allowing the patient to move from their bed, but gives the nurse oversight and ability to check on a patient following bed rail activity.

## 2. Methods and System Design

**Figure 1:**
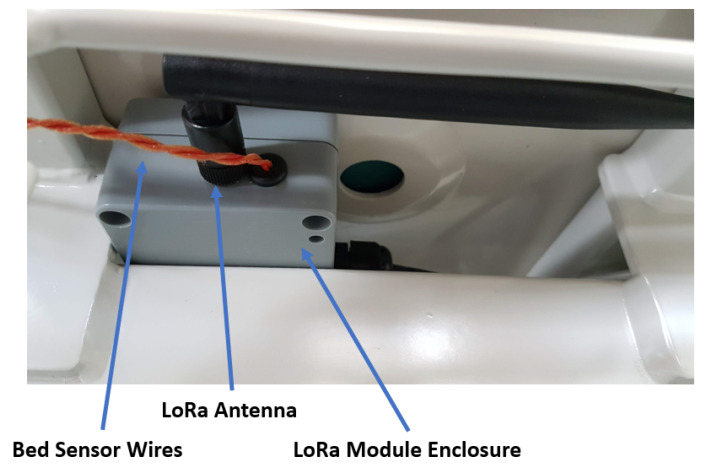
Photograph of wireless LoRaWAN unit.

The overall concept for the LoRaWAN hospital bed wireless system was based around finding a method to allow connectivity and data transmission to occur over long distances inside a hospital using a secure and reliable connection. Wireless technologies such as BLE and WiFi were considered, however, given that beds can travel large distances inside the hospital building, there would be a significant infrastructure requirement to create secured WiFi networks with sufficient coverage. It was considered a poor choice to rely on existing hospital WiFi networks for connectivity, which are already heavily used for general internet and other hospital systems. Adding a secondary method of communication, not reliant on the general infrastructure and centred around a fast installation and minimum capital expenditure, was considered ideal. Using LoRaWAN in this application can produce a robust and flexible system to allow a network of hospital beds to send status and environmental conditions to a central area such as a nurse’s station. A photograph of the unit, installed on a hospital bed, is presented in Figure 1. Reflecting on the COVID-19 crisis, in the future, there is likely to be a growing need to setup new hospital wards quickly, and solutions which minimise infrastructure requirements heavily aid in the ability to rapidly deploy to new locations and setup temporary hospital wards. A smart bed rail using a LoRaWAN wireless system is therefore both an innovative healthcare product and a demonstration of the effectiveness of a LoRaWAN hospital wireless system in allowing connectivity and transmission to occur over long distances using a secure and reliable connection.

Liang et al. showed in 2020 that a concrete building of comparable size to many hospitals (12 floors) could maintain acceptable signal reception at all points of the building from a single LoRaWAN gateway placed at any corner [7]. Saban et al. also showed that a single gateway placed in the corner of a series of large building could service more than one building, and cover all levels [8]. This type of long distance signal penetration makes LoRaWAN perfect for a low cost system installation, allowing a central processing node to manage and receive transmissions from all beds in the hospital.

The key benefits for the use of LoRaWAN in this hospital application are:

- Low power consumption for transceivers allowing long battery backup times [9];
- Long distance and acceptable penetration of common building materials such as concrete [7];
- Reasonable security on par with other established technologies [10];
- Flexibility, with a single gateway allowing many concurrent connections [11];
- Low cost [12].

**Figure 2:**
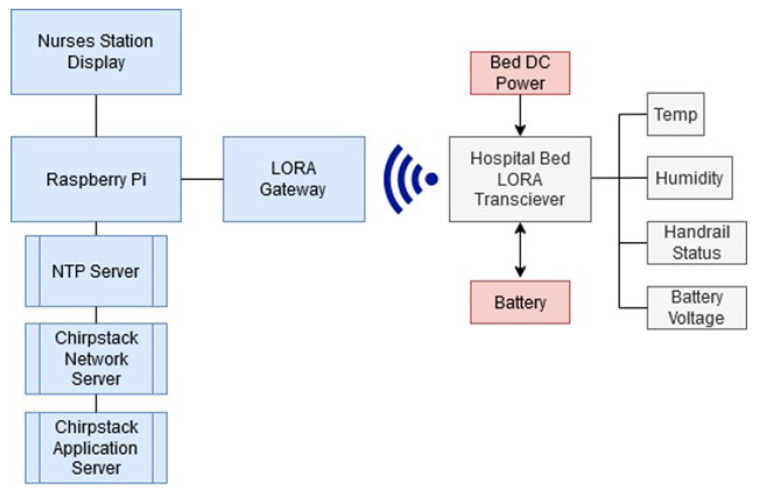
Overview of the system network topology and power inputs for remote node.

Figure 2 shows the hardware layout for the systems network connectivity. A Raspberry Pi is utilised as the main nurses station computational unit. The Raspberry Pi would likely be replaced with a more powerful network server in a real implementation of the system but was sufficient for testing purposes. The remote node is constructed from a Pycom Lopy4 and Sense Shield. It utilises a small external 7dBi omni-directional antenna mounted externally to the node enclosure on the bed (see Figure 1). The remote node’s Sense Shield comes equipped with temperature and humidity sensors as well as integrated battery voltage sensing and control. Consideration is given to the appropriate sizing of the battery for the system and is detailed in Section 3. The following sections outline the design considerations and trade-offs followed by the performance of the prototype system developed.

### 2.1. Offline Operation

A design consideration early on, given the hospital use case, was ensuring connectivity in the event of internet or power outage. Whilst many systems such as Wi-Fi and Bluetooth do not require precise timing, LoRaWAN gateways running the Chirp application stack require the time to be precisely known for time stamping purposes. This process relies on synchronisation with a network time server on the internet to ensure the gateway’s time is correct. Obviously, this is problematic in the event of an internet connectivity loss and causes the gateway to fail in its operation.

**Figure 3:**
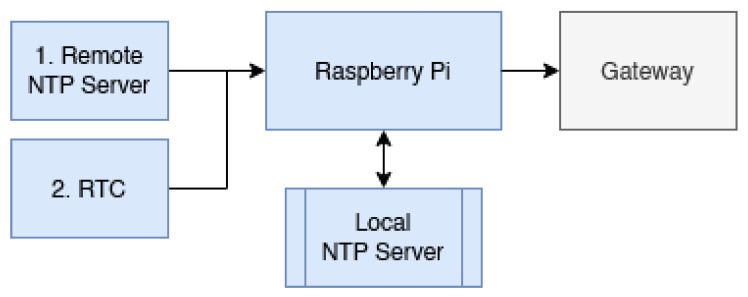
Network time server solution for offline use.

In order to overcome this problem, the system requires its own Network Time Protocol (“NTP”) server to provide the gateway with the current time on request. The problem is further compounded using a Raspberry Pi which does not include a Real Time Clock module. To enable the system to operate in an offline capacity, the system in Figure 3 was developed and integrated into the Raspberry Pi.

As shown in Figure 3, the Raspberry Pi is configured to run its own Network Time Protocol server. The corresponding NTPD configuration file is modified in an unconventional way in order to allow it to maintain its local network segment time sync even when offline. The configuration is set in a cascading manner which typically pulls the network time from a reliable internet server and periodically syncs the real time clock with this value. In the event that the internet server is unavailable, the configuration file is modified in order to “trick” it into believing the real time clock module is providing the network time in an authoritative way.

This prototype system is not best practice as a network time server should only obtain and distribute the current time from true accurate sources. In an actual deployment of the system, it would be modified to include an alternative clock source such as a GPS or atomic time module which can more accurately store the current time. As a final source of redundancy, however, for this application, using the RTC module, which contains its own internal battery and relies on no external network, is still a positive feature.

Despite challenges with using a Raspberry Pi and indeed any application of using a LoRaWAN network offline, the prototype has proved to be successful in initial testing.

### 2.2. Gateway

Gateways in the LoRaWAN architecture are an essential but somewhat interchangeable segment of the network structure. While different gateway manufacturers produce products which contain various antenna configurations and network tools to manage the gateway, they all serve a similar purpose as that of WiFi in allowing remote nodes to connect to the network. The critical difference with that of WiFi is that a node cannot connect to the network unless it is authorised and verified by an application server. Thus, the gateway alone will not provide connectivity to the nodes as they must be externally managed, in this case through the Chirpstack application running on the Raspberry Pi.

LoRaWAN is a system which fundamentally is more focused on providing secure node connectivity to devices across the internet. Thus, local implementations such as the one developed in this solution are less common.

**Figure 4:**
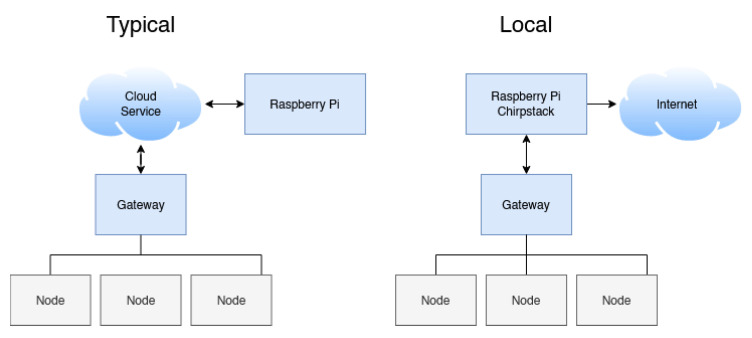
Comparison of the typical LoRaWAN system and prototype hospital bed node system.

Figure 4 displays the differences between a typical LoRaWAN implementation and the one developed for this solution. It is clear that, in the typical example, any disruption to the internet connection will render the nodes unable to communicate with the nurse’s station which is unacceptable in this particular use case.

### 2.3. IoT Architecture

The architecture for the IoT system is based entirely around an open-source implementation. It features the following systems running concurrently on the Raspberry Pi and integrated to allow databasing and information management:

- Chirpstack;
- Mosquito MQTT Server;
- Node Red;
- Influx DB;
- Grafana.

Data obtained from the sensors on the remote bed node are formatted into bytes for transmission which are then assembled into a prescribed message payload. LoRaWAN devices require the user to encode messages and then decode messages at the other end. When the gateway receives a new message, the application server (Chirpstack) passes the message payload to the MQTT server. From here, Node Red receives the new message and performs a Javascript parsing function to decode the long sequence of bytes into several shorter numerical values and assign those values to human readable variables. These values are stored in an Influx DB database and then recalled by Grafana for display. The user at the “nurses station” has access to the current status of the sensors as well as historical values for determining when system changes occurred.

### 2.4. Wireless Technology Suitability

When we think about the hospital setting and its unique requirements as a use case, there are several wireless technologies which could be applied for the bed connectivity system. Displayed in Table 1 is a comparison table showing the typical range, power, and bit rate trade-offs between different wireless technologies.

The more typical and popular wireless technologies, i.e Bluetooth and WiFi, are less than ideal in a hospital setting. Bluetooth is more suited to low range communication at a distance of several meters [12], and WiFi requires extensive infrastructure investment to allow connectivity throughout an entire building. While most hospitals already have this WiFi connectivity, it is heavily used for normal internet traffic and data transfer. Burdening this system with hundreds, if not thousands, of IoT enabled beds would reduce speed and reliability of the system [14]. In the event of a power outage or internet connectivity dropout, the units would be rendered non-operational.

The mesh network protocols such as Z-Wave and Zigbee show promise for the hospital setting, which usually has beds in close proximity. However, they cannot be engineered as a solution in the same way. As the beds are mobile and often moved, the mesh network will only remain operational as long as a the maximum transmission distance is kept below the minimum distance to the next node (i.e., 30 m for Z-Wave). A potential situation could arise in which relocating of beds dynamically breaks the mesh network by exceeding this distance. If this was to occur, any beds past the break would be rendered unconnected. This risk was considered too high for these technologies without significant infrastructure investment such as multiple repeaters and gateways around the hospital. To engineer the system as a standalone unit, it was decided to use LoRaWAN as the base wireless transmission protocol. This was due to the extremely long-distance capabilities of the technology and low power consumption. Using LoRaWAN, it is reasonable to expect that a single commercial gateway, well placed in the hospital, could provide connectivity to several thousand beds and the system could be adapted to new environments very quickly. Saban et al. performed thorough testing on the indoor and outdoor transmission quality that can be expected with LoRa modulation. Their experiments showed that, in outdoor urban environments, a distance of 400 m could be achieved with no packet loss. Indoors, a large series of buildings up to four levels was tested with no packet loss [8].

## 3. Power Analysis

This section describes the power considerations for the system. Given the hospital use case, it was important to provide battery backup functionality and have an understanding of how long the system will remain functional during a power outage. Analysis of the transmission and sleep power usage was performed using a Keithley 2460 Source Meter and the results provide a mathematical method to establish correct battery sizing and transmission dwell time to achieve an estimated battery operation time. Ruckebusch et al. showed that there is significant differences in LoRa modem power consumption, with the lowest being half that of the highest in their testing [15]. With that in mind, the testing results we present are only valid for the hardware configuration we used; however, the trend in results can be extrapolated and the reader can make estimations based on the data-sheet for their modem.

### 3.1. Testing Results

A key design consideration for this system was its ability to remain powered when the bed has been unplugged from a general power outlet. This is clearly a common occurrence in hospital beds as they are moved from room to room for various reasons. The system was designed to operate with very little power draw allowing it to remain connected to the network and still provide updates on key metrics. In order to accurately discover the power consumption of the device and allow selection of a suitably sized battery, a number of tests were performed.

The node module was connected to a Keithley 2460 Source Meter and powered using the internal power supply. The units accuracy resolution was set at 500 mA, and the node was programmed to send an update message every 10 s.

Through experimentation with changing the transmission sleep time, it was discovered that, if messages are sent too often, the gateway will drop them and fail to register a new message for a period of time. We were unable to validate the exact periods involved in this behaviour as it appeared erratic; however, it was in the 1–5 s time band for $t_{int}$, and a hypothesis is that this was due to firmware settings, which we could not access on the gateway.

**Figure 5:**
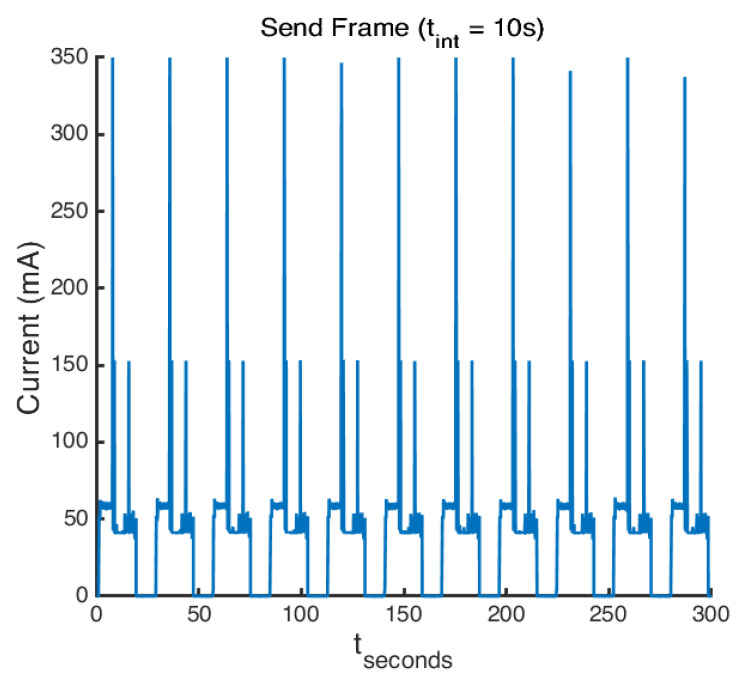
Current consumption over time showing frame transmission peaks. Deep sleep mode enabled between transmissions with a sleep duration ($t_{int}$) of 10 s.

Displayed in Figure 5 is the current draw (mA) versus time for the duration of the test (5 minutes). Interesting spikes can be seen in the plot which shows the activity of the node during its transmission period. The initial peak represents the modem preparation and joining, followed by a large spike during the initial data transmission, and then a moderate peak while the modem waits for its two receive windows to close. It can be seen that the current during sleep mode falls to near zero; with the actual consumption being in the *u*A range, this was subsequently measured in a later test.

From analysis of the data, key system metrics were derived, which provided the average power consumed to send a single frame. Further analysis was performed on the peak message current spikes, which was averaged and found to be 354.94 mA for the 11 frames sent.

**Figure 6:**
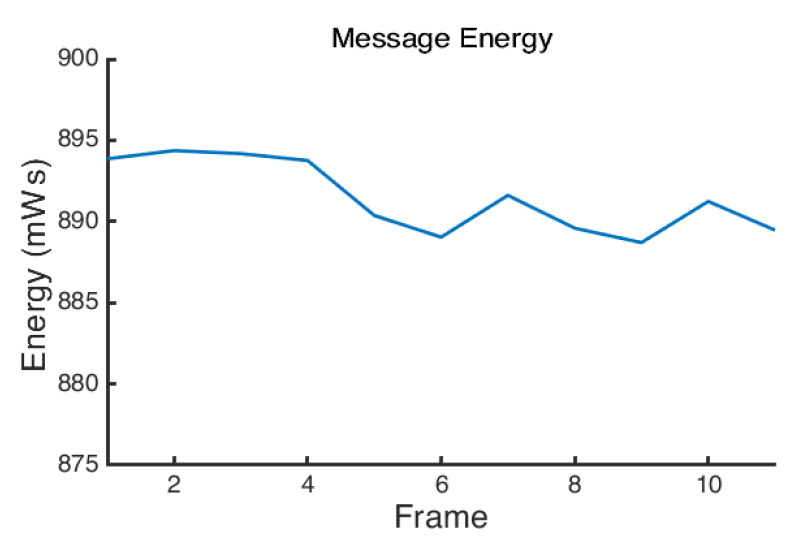
Energy consumed during individual frame transmission, obtained from integration of main node current capture.

The message energy required to send a single frame was averaged and found to be 891.47 mWs, as shown in Figure 6. This is the energy required to send a single frame from the LoRaWAN node. However, the LoRaWAN node spends the majority of its time in deep sleep mode, which uses significantly less power. In order to calculate the size of the battery required to operate the device and its expected power consumption over time, we must also have an average power figure for its consumption during sleep.

The same apparatus was used in order to measure the sleep current; however, this time, the accuracy resolution was set in the *u*A range to allow accurate readings of the very low power consumption during sleep. In order to ensure reliability of the sleep current figure, a separate program was loaded onto the device, which would send a single message and then sleep for a longer duration. The sleep current was then averaged over a larger period of time. It was found to be quite reliable with very little variation even when measuring within the *u*A range. Displayed in Table 2 are the results from this and the previous testing.

### 3.2. Calculations

With the parameters found in the previous section, there was sufficient information available to perform an estimation of the power required to operate the node in different conditions.

In the calculations shown below, we first define $t_{int}$ as the transmission period or “dwell time”, and $E_{battery}$ as the energy stored in the battery. We use the parameters shown in Table 2 to provide the required energy consumed during transmission (at 5 V nominal system voltage). With these values, it is possible to estimate the expected run time on battery power as follows:

(1) $$E_{message}=\frac{86400}{t_{int}}E_{frame}$$

is the energy per day to send a message at interval $t_{int}$.

(2) $$E_{sleep}=I_{sleep}\;V_{ref}\;86400-\frac{86400\;t_{message}}{t_{int}}$$

is the energy consumed during sleep.

(3) $$t_{days}=\frac{E_{battery}}{E_{message}+E_{sleep}}$$

is the battery life estimation. Using a baseline value of $t_{int}=30$ s and substituting the experimentally derived figures, we arrive at a fixed equation for this application:

(4) $$t_{days}=\frac{E_{battery}}{2569.1}.$$

The battery chosen for the prototype unit based on easy availability and size was a 3.7 V 2400 mAh lithium pouch cell. This battery contains 31,968 Ws of available stored energy. Substituting this energy capacity into Equation (4) yields a final result of 12.44 days power at $T_{int}=30$ s.

It must be noted that this is only an estimation and of course the number of messages sent will affect this capacity greatly. The battery will also be subject to aging due to cycle and calendar degradation. The maximum discharge rate of the battery can be easily calculated based on the 355 mA current spike recorded; this corresponds to a 0.15 C max discharge rate. Gao et. al demonstrated that, for lower discharge rates, there is less degradation to be expected for lithium ion batteries [16]. They showed that, for a 0.5 C discharge rate, based on their testing, one could expect a 15% degradation after 1000 cycles. All values associated with this battery life estimation are only an attempt to provide context to different use cases for development of different products and cannot replace real testing of the final prototype.

## 4. Conclusions

The paper presented an atypical offline based LoRaWAN application for use in hospital settings where the ability to maintain network connectivity during internet connection disruption is paramount. Existing research indicates that a single LoRaWAN gateway could be able to cover an entire modern hospital allowing minimal infrastructure cost to implement the device, or for application in a rapidly deployed field hospital. A high quality prototype was manufactured and tested proving the system works as intended and provides bed data to the nurses station reliably, including operation under battery backup. The power consumption of the system under different transmission intervals was tested allowing appropriate battery sizing for different applications to be specified accurately. The advantage of a system such as this in a hospital context is also the myriad of ways in which other systems could be added to the augment it. The Grafana dashboard built for this application can be interfaced on the local network side using MQTT, InfluxQL, HTTP, or through installing further applications—essentially any modern web based protocol. Future developments could include the application of additional sensing to the hospital bed, such as weight sensors, a Bluetooth heart rate monitor and atmospheric quality sensing. Given the mobility of modern hospital beds, a long range wireless radio system such as the one developed can allow crucial detection of patient environment and personal parameters, which can be utilised for advancing patient care as well as investigation into incidents to provide useful time-based metrics.

## Figures and Tables

**Table 1:** Wireless technology comparison of key features. Adapted from: Security and Privacy Issues in IOT Things [13].

	Bluetooth LE	ZigBee	WiFi	Wi-Max	LoRaWAN	LTE	Z-Wave
Standards	IEEE 802.15.1	IEEE 802.15.4	IEEE 802.11 ah	IEEE 802.16	IEEE 802.15g	3GPP	Z-Wave Alliance
Network Types	P2P	Mesh	WLAN	MAN	LPWAN	GERAN	Mesh
Power Concumption	Very Low	Low	High	Medium	Very Low	Medium	Very Low
Data Rate	1 Mbps	0.25 Mbps	Up to 7000 Mbps	70 Mbps	250 kbps	0.1–1 Gbps	0.1 Mbps
Range	35 m	10–100 m	1 km	50 km	100 km	28 km/10 km	30 m
Spectrum	2.4 GHz	2.4 GHz	2.4–5 GHz	2–11 GHz	868–915 MHz	700–2600 MHz	908.42 MHz

**Table 2:** Numerical results from power analysis of the prototype.

Parameter	Measurement	Unit
Sleep Current	9.81	uA
Frame Energy	891.47	mWs
Mean Message Time	18.24	s

## Data Availability

https://github.com/solomonould/lorahospitalbed (accessed on 18 July 2022).
